# Women’s exposure to intimate partner violence and its association with child stunting: findings from a population-based study in rural Rwanda

**DOI:** 10.1080/16549716.2024.2414527

**Published:** 2024-10-16

**Authors:** Jean Nepo Utumatwishima, Ingrid Mogren, Kristina Elfving, Aline Umubyeyi, Ali Mansourian, Gunilla Krantz

**Affiliations:** a Department of Public Health and Community Medicine, Sahlgrenska Academy, University of Gothenburg, Gothenburg, Sweden; b College of Medicine and Health Sciences, School of Public Health, University of Rwanda, Kigali, Rwanda; c Department of Clinical Sciences, Obstetrics and Gynecology, Umea University, Umeå, Sweden; d Department of Physical Geography and Ecosystem Science, Lund University, Lund, Sweden

**Keywords:** Child stunting, intimate partner violence, women, Rwanda, child growth

## Abstract

**Background:**

Child stunting is a significant challenge for most districts in Rwanda and much of sub-Saharan Africa and persists despite multisectoral efforts. There is a notable lack of population-based studies examining the correlation between violence against women and child stunting in Rwanda.

**Objective:**

We aimed to investigate the association between Rwandan women’s exposure to intimate partner violence (IPV) and child stunting in children under 3 years of age.

**Methods:**

In December 2021, a population-based cross-sectional study was conducted in the Northern Province of Rwanda, including 601 women and their children <3 years of age. The World Health Organization (WHO) Women’s Health and Life Experiences Questionnaire for IPV research was utilized. Child stunting was assessed using the WHO criteria for low height for age. Multivariable logistic regression was used to examine the association between IPV and child stunting before and during pregnancy.

**Results:**

Of 601 women, 47.4% (*n* = 285) experienced any form of IPV during pregnancy. The prevalence rates of the types of IPV associated with child stunting varied: 33% for psychological, 31.4% for sexual, and 25.7% for physical violence. Exposure to physical violence before pregnancy and sexual violence during pregnancy was associated with higher odds of child stunting; the adjusted odds ratios were 1.29 (95% CI, 1.01–2.03) and 1.25 (95% CI, 1.04–2.01), respectively.

**Conclusion:**

Women’s exposure to physical and psychological violence is associated with an increased risk of child stunting. Urgent targeted interventions and support systems are needed to address the complex relationship between women’s exposure to IPV and adverse effects on child growth.

## Paper context

- **Main findings:** Intimate partner violence against women correlates with child stunting, exhibiting varying prevalence rates for different forms of violence occurring both before and during pregnancy.
- **Added knowledge:** This study enriches the current literature by incorporating a temporal dimension of intimate partner violence occurring before and during pregnancy, while also shedding light on contextual factors within rural African settings where violence against women persists.
- **Global health impact for policy and action:** Improved comprehension of the types, frequency, and timing of women’s exposure to intimate partner violence is crucial for designing and implementing interventions aimed at mitigating the adverse impacts of violence on child growth, particularly in resource-constrained countries such as Rwanda.

## Background

Poor nutrition remains a public health challenge in low- and middle-income countries, mainly in sub-Saharan Africa and southern Asia, primarily affecting children <5 years of age [1]. Undernutrition has adverse impacts on the cognitive and physical health of children [2]. In addition to the short- and long-term consequences for children’s health, undernutrition has a negative impact on the productivity of affected countries due to lower educational performance, cognitive deficits, and reduced productivity in adulthood [3,4].

Child stunting is characterized by low height for age [5]. It arises from persistent or recurring undernutrition and is commonly linked to factors such as poor maternal health and nutrition, resulting in poor caretaking of the child, inappropriate feeding and care during early childhood resulting in frequent illnesses and symptoms of depression [6,7]. In addition to the obvious factors causing underweight children, there are a number of reasons related to overall life circumstances that have not been fully addressed. Among the various forms of undernutrition, stunting is particularly inhibitory, significantly impeding children from attaining their full physical and cognitive potential [3].

From 2010 to 2020, Rwanda effectively reduced the stunting rate by about 11% over a decade and 5% over 5 years [8]. However, the prevalence remains increased compared with the Africa-wide average and the national objective of a 4% annual decrease in stunting [7]. Despite efforts by the Rwandan government to combat undernutrition in children <5 years of age, the national average rate of stunting remained higher at 33.5% in 2020 [7,9]. Prevalence rates are higher in the Western and Northern provinces, reaching 40% and 41%, respectively. The rate in the Northern province is the same as that documented in Rwanda before 2015 [9,10].

Poor maternal health is a notable factor associated with child stunting [5,11]. Several initiatives have investigated the correlation between poor maternal health and child stunting using demographic health survey (DHS) data. It has been reported that a significant proportion of women with stunted children had experienced one or more forms of intimate partner violence (IPV) [12]. IPV affects women’s rights and health on a global scale [13]. In 2013, the World Health Organization (WHO) conducted a multi-country study that revealed that over a third of women had experienced sexual and/or physical violence from an intimate partner [14]. In a 2018 global estimate study, the highest prevalence of lifetime exposure to intimate partner physical and/or sexual violence among ever-partnered women aged 15–49 years was found in central sub-Saharan Africa (32%) and Oceania (29%), followed by eastern sub-Saharan Africa (24%) and south Asia (19%) [15]. Beyond the impact on women in general, the violence extends to women during pregnancy, which can lead to detrimental effects on their health and may also affect the foetus [16]. Violence during pregnancy is linked to common mental disorders, postpartum depression, obstetric complications, insufficient patient-related prenatal care, increasing risk of premature delivery, low birth weight of the child and poor breastfeeding practices [16–20]. IPV may constrain women from providing sufficient nutrition for their children, possibly stemming from a psychological lack of interest in childcare responsibilities [21]. Similar to other sub-Saharan countries, IPV is highly prevalent in Rwanda and has adverse effects on attendance for antenatal care [22]. Several studies conducted in Rwanda examining the prevalence of IPV among pregnant women revealed an estimated rate of 35.1% for physical IPV [23–26].

In Rwanda, a discernible research gap exists regarding the temporal aspects, i.e. before and during pregnancy and the frequency of mild or more severe violent acts directed towards women and its correlation with child stunting. This study aims to fill this gap, enhancing understanding of the risk factors associated with child stunting in Rwanda. This study is an integral component of a sustained collaboration between the University of Rwanda and five Swedish universities, constituting the Undernutrition Program, an interdisciplinary initiative centred on children and their mothers. Its objective is to scrutinize the persistent issue of stunting in the Rwandan Northern Province by combining theories and methodologies from diverse scientific disciplines, including agriculture/veterinary science, public health, paediatrics, nutrition, and geographical information systems, with an innovation component.

This specific investigation aimed to investigate the association between child stunting and IPV before and during pregnancy and milder and more severe acts, thereby making a substantial contribution to the ongoing research efforts in this field.

## Methods

### Study design, study population and sample size

This cross-sectional, population-based study conducted in the predominantly rural Northern Province included women with children aged 1 month to 3 years. The sample size was determined considering the estimated prevalence of child stunting in Rwanda (38%). The aim was to achieve 4% precision and a 95% confidence level in investigating child stunting as the main study outcome. After calculations, the required sample size was 630 women with children aged 1 month to 3 years.

The sampling process involved a multi-stage random selection procedure. Initially, 186 of the 2743 villages in the Northern Province were randomly selected using a geospatial grid system. Within each selected village, community health workers (CHWs) identified households with women who had children aged between 1 month and 3 years to be interviewed. If a woman was not available in the initially selected household, the nearest household with an eligible woman was approached. Due to reasons such as congenital malformations in children or severe illness, data required for specific analyses (weight and height) were not collected in 29 households, resulting in a participation rate of 95.4% (*N* = 601).

## Data collection procedure

During data collection, only one woman per household was interviewed to ensure the safety and confidentiality of the participants. Information about their partners was provided by the women themselves. Data collection was carried out between November and December 2021, using a structured, tablet-based questionnaire administered by an interviewer. This questionnaire included items on the sociodemographic and psychosocial characteristics of the household, items related to physical, sexual violence and psychological abuse directed at women and anthropometric measurements of children.

The Women’s Health and Life Experiences Questionnaire developed by the WHO was used (15). This questionnaire is a validated instrument designed for studying different forms of IPV. This tool has demonstrated cross-cultural validity and has a documented history of successful utilization in Rwanda and other countries worldwide [23,27,28].

The questionnaire was translated into Kinyarwanda, one of Rwanda’s official languages spoken by all Rwandans. It was then adapted into a tablet-based tool using the iMSEP (internet-based information management system for environmental protection and disaster risk management) platform [29]. This allowed for digital data collection and storage within a centralized cloud-based database. The survey was conducted by a team of proficient interviewers, comprising six PhD students, eight registered nurses, and one post-doctoral researcher, supervised by both Rwandan and Swedish supervisors. The team underwent 5 days of rigorous pre-field training to ensure accuracy and efficiency. A pilot of the questionnaire was conducted over 2 days in one village in the Northern Province.

The University of Rwanda, College of Medicine and Health Sciences, School of Public Health (SPH) spearheaded the implementation of the survey.

### Measurements

#### Dependent variables

Child stunting was defined as low height for age, indicated by measurements below − 2 standard deviations from the median of the international growth reference curve [30]. The height and weight distribution of children aged 1 month to 3 years was compared with the WHO Child Growth Standards reference population [30]. The data on weight, height and age were then used to compute height-for-age z scores, referencing the WHO’s anthropometric standards [31]. The child stunting variable was dichotomized into stunted and not stunted.

Standard procedures were used to measure the child’s weight and height. The ShorrBoard measuring board was used to measure the length of children under 2 years of age in a recumbent position and to measure the height of children aged 2 years and older in a standing position. Weight measurements were obtained using SECA scales featuring a digital display (model number SECA 874). The children were then categorized based on whether they exhibited stunting or not.

#### Independent variables

IPV was assessed by evaluating exposure to physical violence (six items in the WHO questionnaire), sexual violence (three items) and psychological abuse (four items) [14]. Participants were asked to specify if they encountered the same acts once, between two and three times, or three or more times to discern the severity, frequency and temporal patterns of violence before and during pregnancy. Subsequently, composite measures were generated for each category of violence, reflecting exposure both before and during pregnancy. Women reporting any instance/item of violent acts were categorized as exposed to that particular form of violence. This approach aimed to capture a comprehensive understanding of the prevalence and dynamics of violence against women in the specified time frames [32].

#### Sociodemographic variables

Data were recorded for the women and their partners. Their age was initially recorded as a continuous variable and later grouped into two categories (<35 years and ≥35 years). Their educational attainment was initially categorized as incomplete primary school, complete primary school, or secondary school and above. These categories were further grouped as having completed primary school or below or having completed secondary school or above. This reclassification was based on the general educational attainment in Rwanda, where >50% of Rwandan youth have completed only primary school, and >30% have completed secondary school or above. The occupations for both women and their partners were categorized into civil servants, skilled workers, non-skilled workers, and students.

These categories were aggregated into two groups: skilled workers including students, civil servants, and other skilled workers, and non-skilled workers. The monthly household income in Rwandan Francs was divided into two categories: income less than or equal to 36,000 RWF (approximately 30 USD) and more than 36,000 RFW or more.

Women’s and partners’ alcohol drinking habits included the frequency of drinking (daily, weekly, many times during the month, seldom, or never). Alcohol drinking habits were categorized into two groups: those who drank less or never drank at all, and those who drank many times. Apart from the monthly household income, the economic activity within households was assessed by examining the presence of two essential amenities: households with access to electricity and households with access to improved toilet facilities. Toilet quality was assessed based on the WHO definition, categorizing it as either improved (including flush systems, pit latrines with slabs, composting toilets) or non-improved (including shared facilities, open pits, bucket latrines, open defecation) [33].

### Statistical analysis

The prevalence of and frequency of exposure to different acts of violence were estimated using counts (n), percentages of exposed women (%), and the number of violent incidents in different time periods, i.e. before and during pregnancy. Associations between various forms of IPV directed at women before and during pregnancy and child stunting were explored using multivariable logistic regression models. Potential confounding variables were identified through a combination of statistical significance in bivariable analyses and results drawn from previous IPV studies in Rwanda (28). Adjustment was applied for the woman’s age, partner’s age, education levels of both partners, occupation, partner’s alcohol consumption habits, and household access to electricity. *p* values <0.05 were considered to be statistically significant. Measures of association were presented as odds ratios (ORs) with their corresponding 95% confidence intervals (CIs). All analyses were conducted using STATA 17 SE (College Station, Texas, USA).

### Ethical considerations

Participation in the study was voluntary for all selected women; children <3 years of age who were the offspring of the selected women were considered mandatory participants after consent from the mother. The participating children underwent examinations to assess their overall health, and anthropometric measurements were taken to evaluate their growth status. No remuneration was provided for their involvement in the study. Before the interviews, the interviewer thoroughly explained the content of the questionnaire, assured participants of the confidentiality of their responses and emphasized their freedom to withdraw from the study at any point during or after the interview.

The interviews were conducted privately between the participant and the interviewer. In the event of an interruption, interviewers were trained to either conclude the interview or shift the discussion to less sensitive topics until privacy could be ensured. All participants provided written and signed consent. In addition, if any participant required access to healthcare services during the interview, the team facilitated their transportation to the appropriate location. The research protocol and study instruments received approval for scientific and ethical integrity from the Institutional Review Board (IRB) at the University of Rwanda, College of Medicine and Health Sciences, as indicated by approval notice no. 181/CMHS IRB/2021. Approval was also granted by the National Health Research Committee under protocol no. NHRC/2020/PROT/047.

## Results

### Sociodemographic characteristics of the women and their exposure to IPV

The prevalence of physical and psychological violence was significantly higher among women under 35 years of age compared to those aged 35 years and older (54.3% vs. 45.7% and 56.8% vs. 43.2%, respectively), with p-values of 0.02 and 0.03. Women with primary education or below exhibited higher rates of physical violence (n = 123; 87.9%) and psychological violence (n = 166; 83.4%) than those with secondary education or more (physical violence: n = 17; 12.1%; psychological violence: n = 33; 16.6%), with *p* values <0.01 and <0.03, respectively. Women reporting partners with primary education or below experienced markedly higher rates of exposure to physical violence (n = 106; 90.6%), sexual violence (n = 82; 90.1%), and psychological violence (n = 151; 87.8%) compared with those with partners with secondary education or more (n = 131; 21.8%). Specifically, when physical violence against women stratified by their partner’s occupation was examined, significant disparities emerged. Women reporting partners with non-skilled occupations experienced a markedly higher prevalence of physical violence (n = 124; 96.9%) than those with partners in skilled occupations (n = 4; 3.1%; p < 0.01). Regarding alcohol drinking habits, partners (n = 340; 60%) were reported to drink more than women (n = 157; 26.3%). Women whose partners engaged in frequent alcohol consumption (drinking more times in a month) exhibited significantly higher prevalence rates of physical violence (71.0%; n = 98), sexual violence (n = 72; 67.9%) and psychological violence (n = 139; 70.9%). Women residing in households lacking basic amenities, such as access to electricity and improved sanitation, demonstrated a higher prevalence of both physical and sexual violence (Table 1).

**Table 1.** Sociodemographic characteristics of women and their exposure to intimate partner violence.

Variables	Overall, n (%)	Types of intimate partner violence, n (%)								
		Physical violence			Sexual violence			Psychological violence		
		No	Yes	*p* value*	No	Yes	*p* value*	No	Yes	*p* value*
Total (women)	601	371 (72.6)	140 (27.4)		408 (78.8)	110 (21.2)		319 (61.6)	199 (38.4)	
*Age (woman)*										
<35 years	318 (62.2)	242 (65.2)	76 (54.3)	0.02	252 (61.8)	70 (63.6)	0.72	211 (66.1)	113 (56.8)	0.03
≥35 years	193 (37.8)	129 (34.8)	64 (45.7)		156 (38.2)	40 (36.4)		108 (33.9)	86 (43.2)	
*Age (partner)*										
<35 years	280 (49.8)	177 (49.6)	62 (45.9)	0.58	193 (49.2)	49 (46.2)	0.58	158 (52.0)	87 (44.6)	0.11
≥35 years	282 (50.2)	180 (50.4)	73 (54.1)		199 (50.8)	57 (53.8)		146 (48.0)	108 (55.4)	
*Education (woman)*										
Primary level or below	470 (78.2)	281 (75.7)	123 (87.9)	<0.01	318 (77.9)	91 (82.7)	0.27	241 (75.6)	166 (83.4)	0.03
Secondary level and above	131 (21.8)	90 (24.3)	17 (12.1)		90 (22.1)	19 (17.3)		78 (24.4)	33 (16.6)	
*Education (partner)*										
Primary level or below	406 (79.8)	253 (76.7)	106 (90.6)	<0.01	281 (77.8)	82 (90.1)	<0.01	212 (75.2)	151 (87.8)	<0.01
Secondary level and above	103 (20.2)	77 (23.3)	11 (9.4)		80 (22.2)	9 (9.9)		70 (24.8)	21 (12.2)	
Household income in Rwandan Francs (RWF)**										
<36,000 RWF	500 (84.0)	302 (82.3)	124 (88.6)	0.08	336 (83.2)	96 (88.1)	0.21	264 (83.5)	167 (84.8)	0.71
≥36,000 RWF	95 (16.0)	65 (17.7)	16 (11.4)		68 (16.8)	13 (11.9)		52 (16.5)	30 (15.2)	
*Occupation (woman)*										
Skilled work	16 (2.7)	13 (3.6)	2 (1.4)	0.21	13 (3.2)	2 (1.9)	0.46	9 (2.9)	6 (3.1)	0.91
Non-skilled work	574 (97.3)	350 (96.4)	136 (98.6)		388 (96.8)	105 (98.1)		304 (97.1)	190 (96.9)	
*Occupation (partner)*										
Skilled work	40 (7.7)	31 (9.4)	4 (3.1)	0.02	28 (7.7)	7 (6.9)	0.79	22 (7.9)	14 (7.5)	0.86
Non-skilled work	482 (92.3)	298 (90.6)	124 (96.9)		334 (92.3)	94 (93.1)		255 (92.1)	173 (92.5)	
*Alcohol drinking habits (woman)*										
Drink rarely or not at all	439 (73.7)	274 (74.1)	99 (70.1)	0.45	296 (72.7)	80 (72.3)	0.99	236 (74.2)	142 (71.4)	0.48
Drink many times in a month	157 (26.3)	96 (25.9)	41 (29.3)		111 (27.3)	30 (27.3)		82 (25.8)	57 (28.6)	
*Alcohol drinking habits (partner)*										
Drink rarely or not at all	227 (40.0)	161 (44.8)	40 (29.0)	<0.01	168 (42.3)	34 (32.1)	0.06	146 (47.6)	57 (29.1)	<0.01
Drink many times in a month	340 (60.0)	198 (55.1)	98 (71.0)		229 (57.7)	72 (67.9)		161 (52.4)	139 (70.9)	
*Households with access to electricity*										
Yes	226 (37.8)	156 (42.2)	44 (31.7)	0.03	169 (41.5)	33 (30.3)	0.03	126 (39.8)	75 (37.7)	0.64
No	372 (62.2)	214 (57.8)	95 (68.3)		238 (58.5)	76 (69.7)		191 (60.2)	124 (62.3)	
*Households with access to improved toilets*										
Yes	256 (43.3)	174 (47.4)	45 (32.4)	<0.01	185 (45.7)	35 (32.4)	0.01	141 (44.8)	79 (39.9)	0.28
No	335 (56.7)	193 (52.6)	94 (67.6)		220 (54.3)	73 (67.6)		174 (55.2)	119 (60.1)	

*Pearson’s chi-square test.

**Monthly income in Rwandan Francs; 1000 RWF = 1 USD (October 2022).

### Prevalence of violence against women and their association with child stunting before and during pregnancy

Among the 601 participants, 163 women (27.1%) had stunted children. Before pregnancy, 38.8% (233 women) reported exposure to IPV, whereas during pregnancy, this figure increased notably to 47.4% (285 women), indicating a heightened prevalence of any form of violence during this period. Physical and sexual IPV demonstrated more consistent associations with child stunting. During pregnancy, the presence of all three types of violence was associated with an adjusted OR (aOR) of 1.76 (95% CI, 1.02–3.65). Before pregnancy, 38.8% of women experienced any type of violence. During pregnancy, 47.4% of women experienced any type of violence but with a less clear association with child stunting. Before pregnancy, the aOR for physical violence was 1.29 (95% CI, 1.01–2.03) for child stunting, and during pregnancy, the aOR for sexual violence was 1.25 (95% CI, 1.04–2.01) (Table 2).

**Table 2.** Women’s exposure to intimate partner violence before and during pregnancy and their association with child stunting (*N* = 601).

IPV types	Women exposed to IPV, n (%)	Child stunting, n (%)		Crude OR^a^ (95% CI)	Adjusted OR^b^ (95% CI)
		Yes (*n* = 163, 27.1%)	No (*n* = 438, 72.9%)		
*Any type of violence*					
Before pregnancy (*n* = 601)	233 (38.8)	57 (24.5)	176 (40.2)	1.25 (0.86–1.82)	1.27 (0.82–1.96)
During pregnancy (*n* = 601)	285 (47.4)	79 (27.7)	206 (47.0)	1.06 (0.74–1.52)	0.94 (0.61–1.45)
*Physical violence*					
Before pregnancy (*n* = 511)	140 (27.4)	45 (32.1)	95 (25.5)	**1.39 (1.12–2.13)**	**1.29 (1.01–2.03)**
During pregnancy, *n* = 570)	233 (40.9)	60 (25.7)	173 (41.5)	0.91 (0.62–1.33)	0.80 (0.52–1.24)
*Sexual violence*					
Before pregnancy (*n* = 518)	110 (21.2)	30 (27.3)	80 (21.3)	0.98 (0.61–1.57)	1.18 (0.68–1.98)
During pregnancy (*n* = 570)	153 (26.8)	48 (31.4)	105 (25.2)	**1.36 (1.11–2.04)**	**1.25 (1.04–2.01)**
*Psychological violence*					
Before pregnancy (*n* = 518)	199 (38.4)	49 (24.6)	150 (39.7)	0.82 (0.55–1.22)	0.78 (0.49–1.25)
During pregnancy (*n* = 570)	103 (18.1)	34 (33.0)	69 (16.5)	1.44 (0.91–2.28)	1.70 (0.98–2.94)
*All three types of violence*					
Before pregnancy (*n* = 523)	35 (6.7)	7 (20.0)	28 (80.0)	0.65 (0.28–1.52)	0.73 (0.26–2.02)
During pregnancy (*n* = 574)	51 (8.9)	19 (37.2)	32 (62.8)	**1.69 (1.11–3.08)**	**1.76 (1.02–3.65)**

IPV, intimate partner violence; OR, odds ratio; CI, confidence interval. Values in bold type are statistically significant.

^a^Multivariable logistic regression with 95% confidence interval.

^b^Values are controlled for the woman’s age, partner’s age, education levels of both partners, occupation, partner’s alcohol consumption habits, and household access to electricity.

### Women’s exposure to acts of violence during pregnancy and their associations with child stunting

The women’s exposure to each specific form of physical violence, including being slapped, pushed, shoved, hit, kicked, dragged, beaten up, choked, burnt on purpose, or threatened with a weapon, was higher among women with stunted children compared with those without stunted children. All acts of physical violence showed statistically significant crude ORs for having stunted children except for being choked and burnt on purpose. Exposure to forced sexual intercourse during pregnancy (*n* = 22; 14.9%) indicated a higher risk of having stunted children compared with those not exposed to forced sexual intercourse (*n* = 43; 11.3%) with OR 1.38 (95% CI, 1.21–2.41). Women with stunted children reported psychological violence, such as being insulted or feeling bad about themselves (*n* = 42; 29.6%), being belittled or humiliated in front of others (*n* = 12; 9.2%; OR, 1.64; 95% CI, 1.02–3.43), feeling scared or intimidated by their partner on purpose (*n* = 24; 16.9%) and being threatened about hurting someone they cared about (*n* = 10; 7.0%; OR, 1.72; 95% CI, 1.11–3.89) (Table 3).

**Table 3.** Women’s exposure to acts of intimate partner violence during pregnancy and association with child stunting (*N* = 601).

Acts of violence	Women exposed to violence, n (%)	Child stunting, n (%)		Crude OR (95% CI)
		Yes (*n* = 163, 27.1%)	No (*n* = 438, 72.9%)	
*Physical violence*				
Slapped (*n* = 525)	72 (13.7)	24 (16.8)	48 (12.6)	**1.41 (1.23–2.39)**
Pushed or shoved (*n* = 520)	57 (11.0)	19 (13.4)	38 (10.1)	**1.38 (1.34–2.48)**
Hit (*n* = 519)	47 (9.1)	21 (14.9)	26 (6.9)	**2.37 (1.29–4.37)**
Kicked, dragged, or beaten up (*n* = 518)	39 (7.5)	16 (11.4)	23 (6.1)	**1.99 (1.21–3.89)**
Choked or burnt on purpose (*n* = 516)	15 (2.9)	3 (2.2)	12 (3.2)	0.66 (0.18–2.38)
Threatened with a gun, knife or other weapon (*n* = 514)	13 (2.5)	6 (4.3)	7 (1.9)	**2.32 (1.21–4.34)**
*Sexual violence*				
Forced sexual intercourse (*n* = 528)	65 (12.3)	22 (14.9)	43 (11.3)	**1.38 (1.21–2.41)**
Performed unwilling sexual intercourse due to fear (*n* = 522)	85 (16.3)	24 (16.7)	61 (16.1)	1.04 (0.62–1.74)
Forced to perform degrading or humiliating sexual intercourse (*n* = 517)	14 (2.7)	3 (2.1)	11 (2.9)	0.71 (0.19–2.57)
*Psychological violence*				
Insulted or felt bad about herself (*n* = 524)	144 (27.5)	42 (29.6)	102 (26.7)	1.15 (0.75–1.76)
Belittled or humiliated in front of other people (*n* = 489)	33 (6.7)	12 (9.2)	21 (5.8)	**1.64 (1.02–3.43)**
Scared or intimidated by partner’s purpose (*n* = 523)	74 (14.1)	24 (16.9)	50 (13.1)	1.35 (0.99–2.89)
Threatened to hurt someone she cares about (*n* = 524)	26 (4.9)	10 (7.0)	16 (4.2)	**1.72 (1.11–3.89)**

Values in bold type are statistically significant.

### Frequency of violent acts experienced by women before pregnancy and their association with child stunting

Women who were exposed to physical violence before pregnancy demonstrated a higher prevalence of stunted children than those who were not subjected to such violence, including acts such as slapping, pushing, shoving, hitting, kicking, dragging, beating, and threats with weapons. The odds ratios indicated an increased likelihood of child stunting associated with higher frequencies of various forms of physical violence directed at the woman. A clear association was found between women experiencing physical violence more than three times before pregnancy and having stunted children (OR, 2.20; 95% CI, 1.11–2.37). Moreover, women who experienced forced sexual intercourse more than once demonstrated an association with child stunting (OR, 2.37; 95% CI, 1.23–5.66). Similarly, women who were subjected to sexual intercourse due to fear were associated with child stunting with an odds ratio of 2.28 (95% CI, 1.13–4.64) for those who experienced such incidents once. Insults and negative feelings about oneself did not show an obvious association with child stunting, but women who were exposed to belittlement or humiliation in front of others, especially when this occurred more than three times, demonstrated an increased risk of child stunting (OR, 1.59; 95% CI, 1.02–2.36) (Table 4).

**Table 4.** Frequency of intimate partner violence acts before pregnancy and associations with child stunting (*N* = 601).

Acts of violence	Women exposed to violence, n (%)	Child stunting; n (%)		Crude OR (95% CI)
		Yes 163 (27.1)	No 438 (72.9)	
**Physical violence**				
*Slapped (n = 526)*				
No	408 (77.6)	105 (73.4)	303 (79.1)	1
Yes (1 time)	47 (8.9)	14 (9.8)	33 (8.6)	1.22 (0.63–2.37)
Yes (2–3 times)	34 (6.5)	8 (5.6)	26 (6.8)	0.89 (0.39–2.02)
Yes (>3 times)	37 (7.0)	16 (11.2)	21 (5.5)	**2.20 (1.11–2.37)**
*Pushed or shoved (n = 520)*				
No	450 (86.5)	118 (83.7)	332 (87.6)	1
Yes (1 time)	20 (3.8)	8 (5.7)	12 (3.2)	1.88 (0.75–4.70)
Yes (2–3 times)	20 (3.8)	6 (4.2)	14 (3.7)	1.21 (0.45–3.21)
Yes (>3 times)	30 (5.8)	9 (6.4)	21 (5.5)	1.21 (0.54–2.71)
*Hit (n = 520)*				
No	454 (87.0)	119 (83.8)	335 (88.2)	1
Yes (1 time)	22 (4.2)	6 (4.2)	16 (4.2)	1.06 (0.40–2.76)
Yes (2–3 times)	17 (3.3)	4 (2.8)	13 (3.4)	0.87 (0.28–2.71)
Yes (>3 times)	29 (5.5)	13 (9.2)	16 (4.2)	**2.29 (1.17–2.89)**
*Kicked, dragged, or beaten up (n = 522)*				
No	458 (87.9)	118 (83.1)	340 (89.7)	1
Yes (1 time)	25 (4.8)	8 (5.6)	17 (4.5)	1.36 (0.57–3.22)
Yes (2–3 times)	10 (1.9)	3 (2.1)	7 (1.8)	1.23 (0.31–4.85)
Yes (>3 times)	28 (5.4)	13 (9.2)	15 (4.0)	**2.50 (1.15–3.40)**
*Choked or burnt on purpose (n = 516)*				
No	497 (96.3)	133 (94.3)	364 (97.1)	1
Yes (1 time)	8 (1.5)	3 (2.1)	5 (1.3)	1.64 (0.39–6.96)
Yes (2–3 times)	3 (0.6)	1 (0.7)	2 (0.5)	1.37 (0.12–15.21)
Yes (>3 times)	8 (1.6)	4 (2.8)	4 (1.1)	**2.73 (1.67–5.11)**
*Threatened with a gun, knife or other weapon (n = 515)*				
No	500 (97.1)	134 (95.0)	366 (97.9)	1
Yes (1 time)	8 (1.5)	5 (3.6)	3 (0.8)	**4.55 (1.07–10.13)**
Yes (2–3 times)	2 (0.4)	0 (0.0)	2 (0.5)	1
Yes (>3 times)	5 (1.0)	2 (1.4)	3 (0.8)	1.82 (0.30–5.11)
Sexual violence				
*Forced sexual intercourse (n = 531)*				
No	445 (83.8)	120 (81.6)	325 (84.6)	1
Yes (1 time)	15 (2.8)	7 (4.8)	8 (2.1)	**2.37 (1.23–5.66)**
Yes (2–3 times)	30 (5.7)	9 (6.1)	21 (5.5)	1.16 (0.65–2.61)
Yes (>3 times)	41 (7.7)	11 (7.5)	30 (7.8)	0.99 (0.48–2.04)
*Performed unwilling sexual intercourse due to fear (n = 527)*				
No	429 (81.4)	119 (82.1)	310 (81.1)	1
Yes (1 time)	15 (2.8)	7 (4.8)	8 (2.1)	**2.28 (1.13–4.64)**
Yes (2–3 times)	40 (7.6)	7 (4.8)	33 (8.6)	0.55 (0.24–1.28)
Yes (>3 times)	43 (8.2)	12 (8.3)	31 (8.1)	1.01 (0.50–2.02)
*Forced to perform degrading or humiliating sexual intercourse (n = 518)*				
No	502 (96.9)	138 (96.5)	364 (97.1)	1
Yes (1 time)	4 (0.7)	2 (1.4)	2 (0.5)	2.64 (0.37–6.18)
Yes (2–3 times)	6 (1.2)	1 (0.7)	5 (1.3)	0.53 (0.06–4.55)
Yes (>3 times)	6 (1.2)	2 (1.4)	4 (1.1)	1.32 (0.24–7.28)
**Psychological violence**				
*Insulted or made felt bad about herself (n = 527)*				
No	349 (66.2)	98 (68.5)	251 (65.4)	1
Yes (1 time)	44 (8.3)	8 (5.6)	36 (9.4)	0.57 (0.26–1.27)
Yes (2–3 times)	77 (14.6)	22 (15.4)	55 (14.3)	1.02 (0.59–1.77)
Yes (>3 times)	57 (10.8)	15 (10.5)	42 (10.9)	0.91 (0.49–1.72)
*Belittled or humiliated in front of other people (n = 522)*				
No	452 (86.6)	118 (84.3)	334 (87.4)	1
Yes (1 time)	12 (2.3)	4 (2.9)	8 (2.1)	1.41 (0.42–4.79)
Yes (2–3 times)	33 (6.3)	9 (6.4)	24 (6.3)	1.06 (0.48–2.35)
Yes (>3 times)	25 (4.8)	9 (6.4)	16 (4.2)	**1.59 (1.02–2.36)**
*Scared or intimidated by the partner on purpose (n = 522)*				
No	449 (86.0)	122 (86.0)	327 (86.1)	1
Yes (1 time)	14 (2.7)	4 (2.8)	10 (2.6)	1.07 (0.33–3.48)
Yes (2–3 times)	29 (5.6)	7 (4.9)	22 (5.8)	0.85 (0.36–2.04)
Yes (>3 times)	30 (5.7)	9 (6.3)	21 (5.5)	**1.15 (0.51–2.58)**
*Threatened to hurt someone she cares about (n = 524)*				
No	495 (94.5)	128 (90.2)	367 (96.1)	1
Yes (1 time)	8 (1.5)	4 (2.8)	4 (1.0)	2.87 (0.71–6.11)
Yes (2–3 times)	6 (1.1)	4 (2.8)	2 (0.5)	**5.73 (1.04–15.6)**
Yes (>3 times)	15 (2.9)	6 (4.2)	9 (2.4)	1.91 (0.67–5.47)

Values in bold type are statistically significant.

### Violent acts experienced by women during pregnancy and their association with child stunting

Pregnant women who were hit and kicked showed a statistically significant association with child stunting, especially among those who experienced such violence more than three times; the crude OR was 2.48 (95% CI, 1.08–3.56) for being hit and 2.62 (95% CI, 1.13–3.61) for being kicked. Women who experienced forced sexual intercourse more than three times exhibited a statistically significant higher prevalence of child stunting (8.2%) than those not exposed to such violence (6.6%) (OR, 1.30; 95% CI, 1.03–2.66).

## Discussion

This study provides important findings on the prevalence of IPV before and during pregnancy among Rwandan women living in rural areas, and the considerable contribution of all forms of IPV to stunting in their children aged between 1 month and 3 years.

Young women, less educated and living with partners who were reported to have low levels of education and excessive drinking habits were exposed to all types of IPV. Furthermore, child stunting was more prevalent in cases where abuse occurred during pregnancy rather than before pregnancy. However, this is most likely a cumulative effect in women being abused both before and during pregnancy, as their health may already have been compromised upon entering the pregnancy phase [34]. Specifically, child stunting was more frequent in women who were exposed to physical and sexual abuse during pregnancy compared with those who were abused before pregnancy. Women’s exposure to IPV during pregnancy correlates with an increased prevalence of child stunting, particularly in cases involving various forms of physical violence and forced sexual intercourse, aligning with previous research [35–37]. Other factors that seriously affected the rural women’s exposure to IPV were young age, husbands with a low level of education, lack of access to electricity, non-improved toilets and being in unskilled job occupations; i.e. poorer women were more exposed to all forms of violence than women who were better off.

### Effects of IPV on child stunting before pregnancy

Before pregnancy, 38.8% of the women experienced some form of violence, emphasizing the prevalence of IPV in this study population. In this period, the form of IPV mainly associated with child stunting was physical violence, which was linked to partners’ behaviours such as excessive alcohol drinking habits. We hypothesized that partners who were reported to have excessive alcohol drinking habits exerted continuous stress on women by misusing the hard-earned household income. This double burden of violence and poverty exerted on women may cause emotional stress, which may limit their food intake making them underweight, which in turn will influence their children’s birth weight. Both chronic stress, which may lead to depression, and lack of proper nutrition before pregnancy have been found to be associated with child stunting [38,39].

### Effects of IPV on child stunting during pregnancy

In contrast to the prevalence of IPV before pregnancy, the prevalence of any form of violence during pregnancy increased to nearly 50% among women in this study, probably signifying a notable escalation in IPV during pregnancy. Before conception, physical violence was the predominant concern; however, during pregnancy, sexual violence emerged as the form correlating with child stunting. The escalation of sexual violence in rural regions during pregnancy could be linked to certain traditional beliefs that emphasize that engaging in sexual activity is imperative for foetal well-being or successful childbirth, thereby exerting coercive pressures on women to participate against their will [40].

Exposure to all three types of violence during pregnancy showed an increased odds ratio with child stunting. This suggests a cumulative effect on the child of the different forms of violence used against the mother. During pregnancy, the link between IPV and child stunting may be explained by the fact that IPV, as a psychosocial factor, can contribute to conditions such as anaemia and underweight in pregnant women, which in turn may affect foetal growth and positive development [41]. Women exposed to violence often experience mental health conditions such as depression and anxiety to a higher degree than unexposed women [42]. The psychological impact of violence experienced by women during pregnancy can affect the immune responsiveness of the foetus, potentially increasing the susceptibility of children to severe and chronic infections such as pneumonia and diarrhoea [43]. Moreover, IPV may induce psychological stress in women, resulting in a diminished metabolic rate as well as compromised feeding of the child and foetal growth impairment [27,44,45].

### Effects of recurrent IPV acts on child stunting

Various forms of violence inflicted upon pregnant women, including physical acts such as slapping, pushing, hitting, dragging, and threats, were found to have a significant impact on child growth with similar patterns as documented in the literature [46]. In addition, instances of sexual violence even when it occurred only once, such as forcing unwanted sexual intercourse on women, were correlated with child stunting. Sexual IPV can affect birth outcomes through behavioural or physiological responses to stress, including the release of vasoconstrictors or cortisol, which can result in intrauterine growth retardation, and/or release of prostaglandins, which can cause premature contractions and result in premature birth and/or low birth weight children [47]. In addition, forced sexual intercourse before pregnancy has been found to be the precursor of many unintended pregnancies, which result in poor child health outcomes [48]. When acts of physical violence were exerted continuously on women before pregnancy, this was correlated to child stunting later. When the violence starts early in the relationship and continues during pregnancy, the physiological and psychological effects last longer and have a direct impact on child health [49].

### Study strengths and limitations

One significant strength of our study is the meticulous anthropometric assessments of children and IPV surveys of women, both conducted by medical and public health professionals under stringent supervision from both Rwandan and Swedish supervisors. These assessments and surveys adhered closely to WHO standards and protocols.

Although this study provides valuable insights into the prevalence and patterns of IPV among Rwandan women and its links to child stunting, there are inherent limitations that should be acknowledged. The data collected on IPV are based on self-reporting by the participants, which may introduce reporting bias due to underreporting or social desirability bias. The retrospective nature of the study, relying on participants’ recollections of events, might also be susceptible to some recall bias. On the other hand, serious trauma such as being exposed to IPV is seldom forgotten, even if it is difficult to recall the number of events.

The study’s cross-sectional design limits the establishment of causal relationships. However, even if the number of events is difficult to remember, the participating women were asked about events of violence before and during pregnancy, which is before the child was born. The sociodemographic factors considered are not exhaustive; there are other unexplored variables influencing the relationship between IPV and child stunting. In addition, there are other limitations in our study as we did not account for certain important covariates, including substance use, pregnancy status (wanted or unwanted), and the participants’ experiences of child abuse. These factors could potentially influence the relationship between IPV and child stunting, and their exclusion necessitates caution when interpreting the results.

Despite these limitations, the study contributes significantly to the understanding of the dynamics of IPV and their implications for maternal and child health in the Rwandan context.

## Conclusion

This study highlights the high levels of physical, sexual and psychological violence against women in Rwanda, both before and during pregnancy. It further highlights clear associations between women’s exposure to IPV and child stunting in Rwanda. Different types of IPV (i.e. psychological, physical and sexual) were found to contribute differently to the prevalence of child stunting. Notably, exposure to both physical and sexual violence significantly increased the likelihood of child stunting, emphasizing the cumulative impact of multiple forms of IPV.

Urgent targeted interventions and support systems are required to address the multifaceted issue of gender-based violence, particularly by empowering women through education and reinforcing gender-based violence one-stop centres. In addition, incorporating education about violence against women into teachers’ training and raising public awareness about IPV before and during pregnancy are crucial steps. Promoting responsible alcohol consumption, enhancing access to basic amenities and integrating IPV screening into antenatal care services and other maternal and child health programmes are essential components of comprehensive efforts to combat IPV. Addressing boys and young men on the issue of violence against women in schools, through the media, sports clubs and other places where they meet is of utmost importance, especially as violence against women is criminalized in Rwanda.

These recommendations, informed by research findings, offer a comprehensive framework for addressing the challenges of IPV and its detrimental effects on the health and well-being of women and children in Rwanda.
